# Beyond Bevacizumab: An Outlook to New Anti-Angiogenics for the Treatment of Ovarian Cancer

**DOI:** 10.3389/fonc.2015.00211

**Published:** 2015-10-05

**Authors:** Sven Mahner, Linn Woelber, Volkmar Mueller, Isabell Witzel, Katharina Prieske, Donata Grimm, Gunhild Keller-v Amsberg, Fabian Trillsch

**Affiliations:** ^1^Department of Gynecology and Gynecologic Oncology, University Medical Center Hamburg-Eppendorf, Hamburg, Germany; ^2^Department of Oncology, University Medical Center Hamburg-Eppendorf, Hamburg, Germany

**Keywords:** ovarian cancer, anti-angiogenic therapy, multikinase inhibitors, pazopanib, trebananib, cediranib, nintedanib

## Abstract

In addition to the monoclonal vascular endothelial growth factor (VEGF) antibody bevacizumab, several alternative anti-angiogenic treatment strategies for ovarian cancer patients have been evaluated in clinical trials. Apart from targeting extracellular receptors by the antibody aflibercept or the peptibody trebananib, the multikinase inhibitors pazopanib, nintedanib, cediranib, sunitinib, and sorafenib were developed to interfere with VEGF receptors and multiple additional intracellular pathways. Nintedanib and pazopanib significantly improved progression-free survival in two positive phase III trials for first-line therapy. A reliable effect on overall survival could, however, not be observed for any anti-angiogenic first-line therapies so far. In terms of recurrent disease, two positive phase III trials revealed that trebananib and cediranib are effective anti-angiogenic agents for this indication. Patient selection and biomarker guided prediction of response seems to be a central aspect for future studies. Combining anti-angiogenics with other targeted therapies to possibly spare chemotherapy in certain constellations represents another very interesting future perspective for clinical trials. This short review gives an overview of current clinical trials for anti-angiogenic treatment strategies beyond bevacizumab. In this context, possible future perspectives combining anti-angiogenics with other targeted therapies and the need for specific biomarkers predicting response are elucidated.

## Introduction

With implementation of the monoclonal vascular endothelial growth factor (VEGF) antibody bevacizumab to first-line treatment of ovarian cancer patients, the first targeted anti-angiogenic therapy for this indication has demonstrated efficacy and was approved in several countries. Following the results of the ICON7 and the GOG-218 study (first-line treatment) (1, 2), the OCEANS (platinum-sensitive recurrence) (3), and the AURELIA trials (platinum-resistant recurrence) (4), bevacizumab is now available for many therapeutic settings in ovarian cancer. However, as efficacy could only be demonstrated with regard to progression-free survival (PFS) in the target groups, all studies failed to show a reliable effect on overall survival (OS).

Further tailored treatment strategies are still under investigation to improve efficacy and possibly reduce toxicity. Apart from an additional antibody (aflibercept) inhibiting the VEGF pathway further anti-angiogenic targets have been identified to compromise carcinogenesis in ovarian cancer patients. With the angiopoietin cascade a parallel, VEGF-independent signal pathway was detected as a possible target for the novel peptibody trebananib and investigated in clinical trials. In addition, several multikinase inhibitors were developed to interfere with the VEGF receptors and multiple intracellular pathways in addition to the VEGF cascade (e.g., FGF and PDGF), which could be implemented by the introduction of pazopanib, nintedanib, cediranib, sunitinib as well as sorafenib. All these drugs have been studied in clinical trials or are still under investigation. This review gives a focused overview of potential anti-angiogenic treatment strategies beyond bevacizumab and summarizes the current evidence.

## Inhibition of Angiogenesis via the Angiopoietin Pathway

### Trebananib

The peptibody trebananib (AMG386) blocks the connection of the angiopoietins Ang1/Ang2 to the Tie2 receptor and therefore addresses a VEGF independent, parallel anti-angiogenic pathway. Following promising phase II trials (5), trebananib was investigated for recurrent ovarian cancer in the international, double-blind phase III TRINOVA-1 trial in which weekly paclitaxel 80 mg/m^2^ was applied with trebananib 15 mg/kg i.v. weekly or placebo (6). In this trial, 919 patients with recurrent ovarian cancer, a platinum-free interval <12 months and ≤3 prior therapies were included. The trebananib arm had a significantly improved median PFS of 2.8 months [7.2 vs. 5.4 months; hazard ratio (HR) 0.66; 95% CI 0.57–0.77; <0.001] (6). Thus, the study met the primary endpoint, although no improvement of OS was seen (19.3 vs. 18.3 months; HR 0.95; 95% CI 0.81–1.11) (7). Compared to bevacizumab, a different profile of adverse events (AEs) was noted. In general, treatment was well tolerated with reported edema, ascites, and pleural effusions but less traditional VEGF-associated effects (hypertension, proteinuria, thromboembolic events). Although a planned study for patients with platinum-sensitive recurrent disease was not initiated, an additional phase III, double-blind study for first-line treatment comparing chemotherapy of carboplatin/paclitaxel with trebananib in combination with chemotherapy followed by a subsequent weekly trebananib maintenance therapy vs. placebo (AGO-OVAR 18, TRINOVA-3) has completed recruitment and is currently under follow-up.

## Inhibition of Angiogenesis by Targeting Multiple VEGF Proteins

In addition to VEGF, the target of bevacizumab, there are further members of the VEGF pathway that can be addressed by investigational agents possibly influencing angiogenesis.

### Aflibercept

Composed of VEGF binding domains from extracellular regions of the VEGF receptor 1 (VEGFR-1) and VEGFR-2, the fusion protein aflibercept has broad affinity binding VEGF-A, VEGF-B and also the placental growth factor (PlGF) (8). So far, this agent has been studied in phase II trials with relapsed ovarian cancer patients.

Two studies concentrated on patients with platinum-resistant disease and symptomatic malignant ascites with the primary endpoint “time to repeat paracentesis” (9, 10). Both studies could demonstrate a better control of malignant ascites with a reduction of the interval between paracenteses (e.g., 55.1 vs. 23.3 days, respectively; 95% CI 10.6–53.1; *p* = 0.0019) (10) although a survival benefit was not achieved. In a further phase II trial, the dose of either 2 or 4 mg/kg aflibercept every 2 weeks was compared in 294 patients (11). In both arms, the assumed overall response rate (ORR) of >5% could not be reached (0.9 vs. 4.6%) (11). Grade 3/4 adverse were noted with hypertension in up to 27.5%, dyspnea in up to 20%, and proteinuria in up to 9.4% of patients. A higher rate of intestinal perforation was observed although the rates differed significantly between the three trials with 1/16 (6.3%) (9), 3/29 (10.3%) (10), and 3/215 patients (1.4%) (11).

As a consequence of these results with low efficacy, no phase III trials investigating aflibercept have been initiated to date.

## Inhibition of Angiogenesis by Multikinase Inhibitors

Compared to the previous anti-angiogenic agents targeting the extracellular receptors, multikinase inhibitors exhibit their potential via intracellular blockade of different signal transduction pathways.

### Pazopanib

Pazopanib is an oral tyrosine kinase inhibitor targeting three different protein kinases (VEGFR, PDGFR, and c-KIT). This drug exhibits both, anti-angiogenic as well as anti-tumorigenic, effects and was already proven to be effective in renal cell cancer (12). Following promising data in phase II trials (13, 14), a randomized, double-blind phase III study (AGO OVAR 16) with 940 patients was initiated by the Arbeitsgemeinschaft Gynaekologische Onkologie (AGO) for first-line treatment. This trial addressed for the first time a solely anti-angiogenic maintenance therapy. Pazopanib vs. placebo subsequent to standard chemotherapy with carboplatin and paclitaxel was administered orally in a dose of 800 mg. A significant improvement in PFS of 5.6 months for patients in the pazopanib arm was noted (median 17.9 vs. 12.3 months; HR 0.77; 95% CI 0.64–0.91; *p* = 0.002) (15). However, no difference in OS was seen. A significant higher rate of grade 3 or 4 AEs, mainly hypertension (30.8%), neutropenia (9.9%), liver-related toxicity (9.4%), and diarrhea (8.2%), was reported in the pazopanib group. In 33% of patients in the pazopanib arm, treatment was discontinued due to AEs, while this rate was only at 6% in the placebo arm. Currently, pazopanib is still investigated in different phase II studies (e.g., NOGGO-TOPAZ, an ongoing phase II study for patients with platinum-resistant recurrence: pazopanib 400 mg/day orally vs. placebo in combination with topotecan 4 mg/m^2^ weekly).

### Nintenanib

Based on significant benefit for lung cancer treatment and promising results from a phase II study in relapsed ovarian cancer (16, 17), another oral triple angiokinase inhibitor nintedanib (BIBF 1120) targeting VEGFR, PDGFR, and FGFR was studied for first-line therapy of ovarian cancer patients. In the positive prospective, randomized phase III study (AGO-OVAR 12/LUME-Ovar 1) nintedanib 200 mg BID vs. placebo was taken parallel to chemotherapy with carboplatin AUC 5/6 and paclitaxel 175 mg/m^2^ followed by a maintenance phase for a maximum duration of 120 weeks (18). In a total of 1366 included patients, median PFS, the primary endpoint, was prolonged from 16.6 to 17.3 months (HR 0.84; 95% CI 0.72–0.98; *p* = 0.024). Of note, treatment-related toxicity was significantly increased in the nintedanib arm with predominantly hematologic and gastrointestinal AEs (Grad ≥3 22 vs. 2%) (18). So far, no significant effect on OS was noted. Although approval of this multikinase inhibitor is currently not expected, these results add important information to future studies in which patient selection and optimized tolerability will represent important aspects of the study design.

### Cediranib

The oral tyrosine kinase inhibitor cediranib is a potent inhibitor of all three VEGF receptors (VEGFR-1, -2, -3) and c-kit with pronounced selectivity for VEGFR-2. It demonstrated activity in an open-label phase II trial among 46 patients with recurrent disease although the dose of cediranib had to be reduced from 45 to 30 mg/day due to significant toxicity, such as hypertension, fatigue, and diarrhea (19). Based on these results, the randomized, double-blind, placebo-controlled phase III trial ICON6 was initiated to evaluate cediranib in 456 patients with platinum-sensitive recurrent disease (20). Patients were randomized to receive six cycles of carboplatin AUC5 or 6 plus paclitaxel 175 mg/m^2^ with either placebo, cediranib 20 mg/day, followed by placebo (concurrent), or cediranib 20 mg/day, followed by cediranib (concurrent plus maintenance) (20). In this further reduced dosage, the treatment was sufficiently well tolerated during initial toxicity assessment (20).

The first presentation of results demonstrated significantly improved PFS in the cediranib concurrent and maintenance arm compared to placebo (11.4 vs. 9.4 months; HR 0.68; *p* = 0.002) as well as significantly improved median OS (20.3 vs. 17.6 months; HR 0.70; *p* = 0.042) (21). The most common cediranib-related AEs included diarrhea, nausea, and fatigue. Although final publication of results is still pending, ICON6 seems to be the first trial with targeted therapies exhibiting a significant effect on OS in an unselected patient cohort (21).

Cediranib raised further attention following the presentation of a randomized phase II trial investigating the combination of cediranib 30 mg daily and the poly(ADP-ribose) polymerase (PARP) inhibitor olaparib 200 mg BID vs. olaparib 400 mg BID alone in 90 women with recurrent platinum-sensitive ovarian cancer and a deleterious germline BRCA1 or 2 mutation. The chemotherapy-free experimental arm of cediranib and olaparib significantly improved PFS from 9.0 to 17.7 months (HR 0.42; 95% CI 0.23–0.76; *p* = 0.005), while OS data are not mature yet (22). As drug-related AEs were more common in the cediranib plus olaparib arm (70% of patients with grade 3 or higher event) than in olaparib monotherapy (11%) further envisaged phase III trials need to account for tolerabilty of this novel combination (22). In this context, the international randomized phase III PAOLA-1 trial was recently initiated by the French Groupe d’Investigateurs Nationaux pour l’Étude des Cancers Ovariens (GINECO) to investigate the combination of chemotherapy, anti-angiogenic therapy and PARP inhibitors in the first-line setting for ovarian cancer patients. Accounting for the approval status in Europe, bevacizumab instead of cediranib was chosen for combination with olaparib and platinum-based chemotherapy.

### Sunitinib

A further multikinase inhibitor targeting VEGF receptors, PDGF receptors, stem cell factor receptor (KIT) and FMS-like tyrosine kinase-3 (FTL3), has also been included in phase II studies for ovarian cancer and recurrent disease (23). Initially, sunitinib as single agent was investigated at a dose of 50 mg daily over 4 weeks of a 6-week cycle, which was adopted to continuous 37.5 mg daily dosing in the second stage of accrual due to higher incidence of ascites or pleural effusions during off-treatment intervals (24). Although sunitinib exhibited modest activity in recurrent platinum-sensitive ovarian cancer, a dosage-dependent response was noted favoring the 50 mg intermittent schedule (24). Common AEs included fatigue, gastrointestinal symptoms, hand-foot syndrome, and hypertension. No gastrointestinal perforation occurred during treatment period (24).

The phase II AGO 2.11 study investigated single-agent sunitinib in 73 patients with platinum-resistant ovarian cancer in which moderate activity was noted. Included patients had received ≤3 prior chemotherapy regimens and were allocated to two treatment arms (arm 1: non-continuous treatment with 50 mg sunitinib daily orally for 28 days followed by 14 days off drug; arm 2: continuous treatment with 37.5 mg sunitinib administered daily). In this trial, patients receiving non-continuous treatment responded better to the systemic therapy regarding PFS [arm 1: 4.8 (2.9–8.1) months; arm 2: 2.9 (2.9–5.1) months], while the median OS [arm 1: 13.6 (7.0–23.2) months; arm 2: 13.7 (8.4–25.6) months] as well as the pattern of AEs did not differ significantly (25). So far, no phase III trial has been initiated.

### Sorafenib

As well as the previous molecules, sorafenib is an oral multitargeted tyrosine kinase inhibitor blocking VEGFR2, VEGFR3, as well as PDGFR beta, Flt-3, and c-kit (26). In addition to these targets, sorafenib has partial inhibitory effects on portions of the RAS/RAF/MEK/ERK signaling pathway, which is known to play a central role in ovarian cancer development, especially in low-grade tumors (26).

In a phase II study of 71 patients concentrating on recurrent ovarian cancer, a modest anti-tumor effect could be demonstrated for sorafenib maintenance treatment at dose of 400 mg twice a day following chemotherapy. However, this impact was achieved at the expense of significant toxicity (27). Comparable results were revealed by a randomized phase II trial of 246 patients with complete remission after first-line chemotherapy in which no significant difference between treatment with sorafenib 400 mg twice a day vs. placebo could be demonstrated for PFS (median 12.7 vs. 15.7 months; HR 1.09; 95% CI 0.72–1.63) (28). Of note, high rates of dose reductions (67.5 vs. 30.1%) and early discontinuations were noted in the sorafenib arm, interfering with the efficacy analysis. The most common ≥grade 3 AEs were hand-foot skin reaction (39.0 vs. 0.8%) and rash (14.6 vs. 0%). The authors concluded that sorafenib, therefore, should not be recommended as maintenance therapy for patients with OC experiencing complete remission (28). Other studies had to be prematurely closed due to low accrual of patients (29). Nevertheless, there are still results of clinical phase II trials for recurrent ovarian cancer pending in which a combination to mono-chemotherapy (e.g., sorafenib plus topotecan, NOGGO-TRIAS) or a chemotherapy-free combination with bevacizumab is being tested.

## Conclusion

Over the past years, different promising therapeutic approaches for anti-angiogenic therapy beyond bevacizumab have been investigated (Table 1). So far, four of these anti-angiogenics (trebananib, pazopanib, nintedanib, and cediranib) were evaluated in phase III clinical trials (Table 2). While pazopanib and nintedanib could already demonstrate to significantly improve PFS of ovarian cancer patients within first-line therapy of two positive trials, the results of a third first-line study investigating trebananib are pending. However, an OS effect could not be observed for anti-angiogenic first-line therapies so far. For recurrent disease, two positive phase III trials investigating trebananib and cediranib gave new insights to find additional, effective anti-angiogenic agents for this group of patients.

**Table 1:** **Overview of phase II studies with anti-angiogenic agents for recurrent disease**.

Author investigated agent	Study design	*n* (rel.)	Results PFS median	Other end points
**Trebananib**				
Karlan et al. (5)	Trebananib 10 mg/kg + paclitaxel 80 mg/m^2^ q1w vs. trebananib 3 mg/kg + paclitaxel 80 mg/m^2^ q1w vs. placebo + paclitaxel 80 mg/m^2^ q1w	161 (1:1:1)	7.2 months (95% CI 5.3–8.1) vs. 5.7 months (95% CI 4.6–8.0) vs. 4.6 months (95% CI 1.9–6.7)	Overall response rate (ORR): 37 vs. 19 vs. 27%
**Aflibercept**				
Colombo et al. (9)	Aflibercept 4 mg/kg q2w (single-arm)	16	59.5 days (95% CI 41.0–83.0)	Median time to repeat paracentesis: 76.0 (95% CI 64.0–178.0) days vs. baseline interval (16.8 days)
Gotlieb et al. (10)	Aflibercept 4 mg/kg q2w vs. placebo	55 (1:1)	6.3 weeks (95% CI 5.9–10.9) vs. 7.3 weeks (95% CI 6.3–14.0)	Mean time to repeat paracentesis: 55.1 (SE 7.3) vs. 23.3 (7.7) days; difference 31.8 days (95% CI 10.6–53.1; *p* = 0.0019)
Tew et al. (11)	Aflibercept 2 mg/kg q2w vs. aflibercept 2 mg/kg q2w	294 (1:1)	–	Overall response rate (ORR): 0.9 vs. 4.6%
**Pazopanib**				
Friedlander et al. (14)	Pazopanib 800 mg daily following complete CA-125 response to initial platinum-based chemotherapy and subsequent rise	36	–	Overall response rate (ORR): 18% in patients with measurable disease at baseline
Pignata et al. (13)	Paclitaxel 80 mg/m^2^ + pazopanib 800 mg daily vs. paclitaxel 80 mg/m^2^	74 (1:1)	Median 6.35 months (95% CI 5.36–11.02) vs. 3.49 months (95% CI 2.01–5.66); HR 0.42 (95% CI 0.25–0.69); *p* = 0.0002	–
**Nintedanib**				
Ledermann et al. (16)	Nintedanib 250 mg BID vs. placebo BID continuously for 36 weeks as maintenance	83 (1:1)	Thirty-six-week PFS rates: 16.3 and 5.0%; HR 0.65 (95% CI 0.42–1.02; *p* = 0.06)	–
**Cediranib**				
Matulonis et al. (19)	Cediranib 45 mg daily, subsequently 30 mg daily (single-arm)	47	–	Overall response rate (ORR): 17% (95% CI 7.6–30.8%)
Liu et al. (22)	Cediranib 30 mg daily + olaparib 200 mg BID vs. olaparib 400 mg BID alone	90 (1:1)	17.7 vs. 9.0 months; HR 0.42 (95% CI 0.23–0.76; *p* = 0.005)	–
**Sunitinib**				
Biagi et al. (24)	Sunitinib 50 mg daily (4 of 6 weeks) subsequently continuous 37.5 mg daily dosing	30	4.1 months	Overall response rate (ORR): 13.3%
Baumann et al. (25)	Sunitinib 50 mg daily (4 of 6 weeks) vs. continuous 37.5 mg daily dosing	73	4.8 months (2.9–8.1) vs. 2.9 months (2.9–5.1)	Overall response rate (ORR): 16.7 vs. 5.4%
**Sorafenib**				
Matei et al. (27)	Sorafenib 400 mg orally BID	71	Patients with at least 6 months PFS: 24% (90% CI 15–35%)	Overall response rate (ORR): 3.4%
Herzog et al. (28) (first-line treatment)	Sorafenib 400 mg BID vs. placebo maintenance in patients with complete remission after first-line chemotherapy	246	Median 12.7 vs. 15.7 months; HR 1.09 (95% CI 0.72–1.63)	–

*Key characteristics and results of published phase II studies for anti-angiogenics beyond bevacizumab in case of recurrent disease*.

**Table 2:** **Overview of phase III studies with anti-angiogenic beyond bevacizumab**.

Trial name (author)	Study design	*n* (rel.)	Results PFS median	Results OS median	Further aspects
**First-line treatment**					
AGO-OVAR 16 [du Bois et al. (15)]	Pazopanib 800 mg orally daily vs. placebo orally daily subsequent to first-line chemotherapy up to 24 months	940 (1:1)	17.9 vs. 12.3 months; HR 0.77 (95% CI 0.64–0.91; ***p*** = **0.002**)	Immature data	Pure maintenance therapy subsequent to first-line chemotherapy
AGO-OVAR 12^a^ [du Bois et al. (18)]	Carboplatin AUC5/6 q3w + paclitaxel 175 mg/m^2^ q3w + nintedanib 200 mg orally BID up to 120 weeks vs. carboplatin AUC5/6 q3w + paclitaxel 175 mg/m^2^ q3w + placebo orally BID up to 120 weeks	911 (2:1)	17.3 vs. 16.6 months; HR 0.84 (95% CI 0.72–0.98; ***p*** = **0.024**)	Immature data	Oral anti-angiogenic therapy parallel to chemotherapy with subsequent maintenance phase
**Recurrent disease**					
ICON6^a^ [Ledermann et al. (21))	Cediranib 20 mg orally daily during platinum-based chemotherapy and followed up to 18 months vs. cediranib 20 mg orally daily during platinum-based chemotherapy and followed by placebo up to 18 months vs. placebo with platinum-based chemotherapy	456 (3:3:2)	11.4 vs. 9.4 months; HR 0.68; ***p*** = **0.0022**	20.3 vs. 17.6 months; HR 0.70; ***p*** = **0.042**	Platinum-sensitive recurrent ovarian cancer; first study with targeted therapy and effect on OS
TRINOVA-1 [Monk et al. (6)]	Paclitaxel 80 mg/m^2^ q1w + trebananib 15 mg/kg q1w vs. paclitaxel 80 mg/m^2^ q1w + placebo q1w	919 (1:1)	7.2 vs. 5.4 months; HR 0.66 (95% CI 0.57–0.77; ***p*** < **0.001**)	19.3 vs. 18.3 months; HR 0.95 (95% CI 0.81–1.11; *p* = 0.52)	Recurrent ovarian cancer with <12 months platinum-free interval

*Key characteristics and results of published phase III studies for anti-angiogenics beyond bevacizumab (first-line vs. recurrence)*.

*^a^So far only presentation at conference, full paper is pending*.

*Statistically significant p values are printed in bold*.

Emerging data suggests that patient selection might represent a central aspect for future studies. Specific histological subtypes and patients mostly benefiting from a distinct treatment regimen need to be identified to avoid unnecessary toxicity and deterioration of quality of life of non-responding patients. Especially regarding maintenance therapies, patient-reported outcomes to assess quality of life more thoroughly as well as interpretation of significant AEs will become progressively relevant.

Combining anti-angiogenics with other targeted therapies to possibly spare chemotherapy in certain constellations, as shown for cediranib and olaparib in BRCA-mutated patients, represents another very interesting future perspective for clinical trials. Identifying drugs with a well-tolerated dosage and dosing schedule, optimal combination partners, and a selection process for patients with expected high response rates will be the major aims for future investigations in ovarian cancer.

## Conflict of Interest Statement

Sven Mahner: research support, honoraria, and travel support: AstraZeneca, Bayer, Boehringer Ingelheim, Jenapharm, GSK, JanssenCilag, Medac, MSD, PharmaMar, Roche, Tesaro, and Teva. Fabian Trillsch: research support, honoraria, and travel support: AstraZeneca, Boehringer Ingelheim, Medac, PharmaMar, and Roche. Volkmar Müller: speaker honoraria from Amgen, Celgene, Eisai, Glaxo Smith Kline, Pierre-Fabre, Roche, and Janssen-Cilag; consultancy honoraria from Roche, Pierre Fabre, Amgen, and Eisai. Isabell Witzel: research support, honoraria, and travel support: Roche. Linn Woelber, Donata Grimm, Katharina Prieske: honoraria/travel support: Jenapharm, Medac Jenapharm, Roche; research funding: GSK, Medac, MSD, PharmaMar, Roche. Gunhild Keller-v Amsberg: Speaker honoraria from AstraZeneca, Bayer, Boehringer Ingelheim, Jenapharm, GSK, JanssenCilag, Medac, MSD, PharmaMar, Roche, Tesaro, and Teva; Consultancy honoraria from Astellas und Sanofi.
